# ﻿Three new *Xylaria* species (Xylariaceae, Xylariales) on fallen leaves from Hainan Tropical Rainforest National Park

**DOI:** 10.3897/mycokeys.86.71623

**Published:** 2022-01-12

**Authors:** Xiao-Yan Pan, Zi-Kun Song, Zhi Qu, Tie-Dong Liu, Hai-Xia Ma

**Affiliations:** 1 College of Forestry, Hainan University, Haikou 570228, China; 2 Institute of Tropical Bioscience and Biotechnology, Chinese Academy of Tropical Agricultural Sciences, Haikou 571101, China; 3 College of Plant Protection, Jilin Agricultural University, Jilin 130000, China; 4 Hainan Institute for Tropical Agricultural Resources, Chinese Academy of Tropical Agricultural Sciences, Haikou 571101, China

**Keywords:** Folicolous fungi, Phylogeny, Pyrenomycetes, Taxonomy

## Abstract

Three new species of *Xylaria* on fallen leaves in Hainan Province of China are described and illustrated, based on morphological and molecular evidence. *Xylariahedyosmicola* is found on fallen leaves of *Hedyosmumorientale* and featured by thread-like stromata with a long sterile filiform apex. Phylogenetically, *X.hedyosmicola* is closely related to an undescribed *Xylaria* sp. from Hawaii Island, USA and morphologically similar to *X.vagans. Xylarialindericola* is found on fallen leaves of *Linderarobusta* and characterised by its subglobose stromata and a long filiform stipe. It is phylogenetically closely related to X.siculaf.major. *Xylariapolysporicola* is found on fallen leaves of *Polysporahainanensis*, it is distinguished by upright or prostrate stromata and ascospores sometimes with a slimy sheath or non-cellular appendages. *Xylariapolysporicola* is phylogenetically closely related to *X.amphithele* and *X.ficicola.* An identification key to the ten species on fallen leaves in China is given.

## ﻿Introduction

Species of *Xylaria* Hill ex Schrank are commonly found throughout the temperate, subtropical and tropical regions of the world, associated with wood, fallen fruits or seeds, fallen leaves or petioles and termite nests (Dennis 1956; Rogers 1986; Rogers and Samuels 1986; San Martin and Rogers 1989; Ju and Rogers 1999; Ju and Hsieh 2007; Fournier 2014). Previous studies on *Xylaria* have dealt primarily with species growing on wood and termite nests (Rogers et al. 2005; Ju and Hsieh 2007; Fournier et al. 2020), but the species diversity and distribution of the genus on other substrates, such as fallen fruits or seeds and fallen leaves or petioles, are still poorly studied (Hsieh et al. 2010; J﻿u et al. 2018). Especially, the study of *Xylaria* species growing on fallen leaves or petioles is far behind those mentioned taxa associated with other substrates and only seven species have been reported on those substrates in China (Dennis 1956; Rogers et al. 1988; Zhu and Guo 2011; Huang et al. 2014, 2015; Ma and Li 2018).

Hainan Province (20°01.04'N, 110°20.95'E) is located in southern China and enjoys a tropical monsoon climate. More than 6036 plant species, 1895 genera and 243 families have been reported in the province (Yang 2015). Different kinds of tropical vegetations (e.g. Moraceae, Euphorbiaceae and Arecaceae) and rainforests are distributed over the vast territory of the province, in which abundant fungi occur (Dai et al. 2009; Dai 2012; Gao and Yang 2016; Cui et al. 2019). Two intensive surveys of xylariaceous fungi were carried out in Hainan province in 2019 and 2020 and about 400 specimens of Xylariaceae were collected. These materials have been carefully studied through both morphological and phylogenetic methods and three new species on fallen leaves were identified. The new taxa are described and illustrated, and an identification key is provided for the 10 known species of *Xylaria* on fallen leaves in China.

## ﻿Materials and methods

### ﻿Morphological studies

Voucher specimens are deposited in the Fungarium of the Institute of Tropical Bioscience and Biotechnology, Chinese Academy of Tropical Agricultural Sciences (FCATAS), Hainan Province, China. Samples for microscopic examination were mounted in distilled water, Melzer’s reagent, India ink or 1% SDS. Microscopic features observation, measurements and photographing were performed by using a Zeiss Axio Imager A2 microscope (Göttingen, Germany) by differential interference contrast microscopy (DIG) and brightfield microscopy (BF). The photographs of stromata, perithecia and ostioles were taken with a VHX-600E stereomicroscope Keyence Corporation (Osaka Japan). The methods of collecting, preservation and identification of the specimens follow Ma and Li (2018).

### ﻿DNA extraction and sequencing

A modified cetyltrimethylammonium bromide (CTAB) extraction kit (Aidlab Biotechnologies, Beijing, China) was employed for total DNA extraction from dried specimens. The ITS region was amplified with the primer pair ITS4 and ITS5 (White et al. 1990) using the following procedure: initial denaturation at 95 °C for 3 min, followed by 30 cycles of 94 °C for 40 s, 55.8 °C for 45 s and 72 °C for 1 min and a final extension of 72 °C for 10 min. The TUB and RPB2 gene region were amplified with primers T1/T22 (O’Donnell and Cigelnik 1997) and fRPB2-5F/fRPB2-7CR (Liu et al. 1999), respectively, using the following procedure: initial denaturation at 95 °C for 3 min, followed by 35 °C cycles of 94 °C for 1 min, 52 °C for 1 min and 72 °C for 1.5 min and a final extension of 72 °C for 10 min (Hsieh et al. 2005). DNA sequencing was performed at BGI tech (Guangzhou, China) and sequences were deposited in GenBank (Table 1).

**Table 1.** Species, specimens and GenBank accession number of sequences used in this study. New species and sequences are set in bold.

Taxon	Substrate / Origin	Specimen No.	GenBank No.			Reference
			ITS	TUB	RPB2	
*Xylariaacuminatilongissima*	termite nests / China Taiwan	HAST 623	EU178738	GQ502711	GQ853028	Hsieh et al. (2010)
*X.adscendens*	wood / Guadeloupe	HAST 570	GU300101	GQ487708	GQ844817	Hsieh et al. (2010)
*X.allantoidea*	trunk / China Taiwan	HAST 94042903	GU324743	GQ502692	GQ848356	Hsieh et al. (2010)
*X.amphithele*	dead leaves / Guadeloupe	HAST 529	GU300083	GQ478218	GQ844796	Hsieh et al. (2010)
*X.apoda*	bark / China Taiwan	HAST 90080804	GU322437	GQ495930	GQ844823	Hsieh et al. (2010)
*X.arbuscula*	bark / China Taiwan	HAST 89041211	GU300090	GQ478226	GQ844805	Hsieh et al. (2010)
X.arbusculavar.plenofissura.	wood / China Taiwan	HAST 93082814	GU339495	GQ478225	GQ844804	Hsieh et al. (2010)
*X.atrodivaricata*	termite nests / China Taiwan	HAST 95052001	EU178739	GQ502713	GQ853030	Hsieh et al. (2010)
*X.badia*	bamboo culm / China Taiwan	HAST 95070101	GU322446	GQ495939	GQ844833	Hsieh et al. (2010)
*X.bambusicola*	bamboo culm / Thailand	JDR 162	GU300088	GQ478223	GQ844801	Hsieh et al. (2010)
*X.berteri*	bark / USA	JDR 256	GU324750	GQ502698	GQ848363	Hsieh et al. (2010)
*X.berteri*	bark / China Taiwan	HAST 90112623	GU324749	AY951763	GQ848362	Hsieh et al. (2010)
*X.betulicola*	leaves of *Betula* / China	FCATAS 750	MF774332	–	–	Ma and Li (2018)
*X.brunneovinosa*	termite nests / China Taiwan	HAST 720	EU179862	GQ502706	GQ853023	Hsieh et al. (2010)
*X.castorea*	wood / New Zealand	PDD 600	GU324751	GQ502703	GQ853018	Hsieh et al. (2010)
*X.cirrata*	termite nests / China Taiwan	HAST 664	EU179863	GQ502707	GQ853024	Hsieh et al. (2010)
*X.coccophora*	wood / French	HAST 786	GU300093	GQ487701	GQ844809	Hsieh et al. (2010)
*X.crinalis*	wood / China	FCATAS 751	MF774330	–	–	Ma and Li (2018)
*X.crozonensis*	bark / France	HAST 398	GU324748	GQ502697	GQ848361	Hsieh et al. (2010)
*X.cubensis*	log / Russian Far East	HAST 477	–	GQ502699	GQ848364	Hsieh et al. (2010)
*X.culleniae*	pod / Thailand	JDR 189	GU322442	GQ495935	GQ844829	Hsieh et al. (2010)
*X.escharoidea*	termite nests / China Taiwan	HAST 658	EU179864	GQ502709	GQ853026	Hsieh et al. (2010)
*X.feejeensis*	bark / China Taiwan	HAST 92092013	GU322454	GQ495947	GQ848336	Hsieh et al. (2010)
*X.ficicola*	fallen leaves and petioles of *Ficusauriculata* / China	HMJAU 22818	MZ351258	–	–	**This study**
*X.filiformis*	herbaceous stem / Iran	GUM 1052	KP218907	–	–	Hashemi et al. (2015)
*X.fimbriata*	termite nests / French West Indies	HAST 491	GU324753	GQ502705	GQ853022	Hsieh et al. (2010)
X.cf.glebulosa	fruit / French West Indies	HAST 431	GU322462	GQ495956	GQ848345	Hsieh et al. (2010)
*X.grammica*	wood / China Taiwan	HAST 479	GU300097	GQ487704	GQ844813	Hsieh et al. (2010)
*X.griseosepiacea*	termite nests / China Taiwan	HAST 641	EU179865	GQ502714	GQ853031	Hsieh et al. (2010)
***X.hedyosmicola***	**fallen leaves of *Hedyosmumorientale* / China Haina**n	**FCATAS 856 (HT)**	**MZ227121**	**MZ221183**	MZ683407	**This study**
***X.hedyosmicola***	**fallen leaves of *Hedyosmumorientale* / China Hainan**	**FCATAS 857**	**MZ227023**	**MZ221184**	MZ851780	**This study**
*X.hypoxylon*	wood / Belgium	HAST 152	GU300096	GQ260187	GQ844812	Hsieh et al. (2010)
*X.hypoxylon*	wood / China Taiwan	HAST 95082001	GU300095	GQ487703	GQ844811	Hsieh et al. (2010)
*X.hypoxylon*	leaf debris / Sweden	CBS 122617	AM993146	–	–	Persoh et al. (2009)
*X.ianthinovelutina*	fruit of *Swietenia* / Martinique	HAST 553	GU322441	GQ495934	GQ844828	Hsieh et al. (2010)
*X.intraflava*	termite nests / China Taiwan	HAST 725	EU179866	GQ502718	GQ853035	Hsieh et al. (2010)
*X.juruensis*	*Arengaengler*i / China Taiwan	HAST 92042501	GU322439	GQ495932	GQ844825	Hsieh et al. (2010)
*X.laevis*	wood / Martinique	HAST 419	GU324746	GQ502695	GQ848359	Hsieh et al. (2010)
*X.leavis*	bark / China Taiwan	HAST 95072910	GU324747	GQ502696	GQ848360	Hsieh et al. (2010)
***X.lindericola***	**fallen leaves of *Linderarobusta* / China Hainan**	**FCATAS 852 (HT)**	**MZ005635**	**MZ031978**	MZ031982	**This study**
***X.lindericola***	**fallen leaves of *Linderarobusta* / China Hainan**	**FCATAS 853**	**MZ005636**	**MZ031979**	**MZ048749**	**This study**
*X.liquidambar*	fruits of *Liquidambarformosana* / China Taiwan	HAST 93090701	GU300094	GQ487702	GQ844810	Hsieh et al. (2010)
*X.longissima*	wood / China	FCATAS 749	MF774331	–	–	Ma and Li (2018)
*X.longissima*	wood / Iran	IRAN 16582 F	KP218906	–	–	Hashemi et al. (2015)
*X.meliacearum*	petioles and infructescence of *Guareaguidonia* / Puerto Rico	JDR 148	GU300084	GQ478219	GQ844797	Hsieh et al. (2010)
*X.multiplex*	wood / USA	JDR 259	GU300099	GQ487706	GQ844815	Hsieh et al. (2010)
*X.muscula*	dead branch / French West	HAST 520	GU300087	GQ478222	GQ844800	Hsieh et al. (2010)
*X.nigripes*	termite nests / China Taiwan	HAST 653	GU324755	GQ502710	GQ853027	Hsieh et al. (2010)
*X.oxyacanthae*	fallen seeds / USA	JDR 859	GU322434	GQ495927	GQ844820	Hsieh et al. (2010)
*X.oxyacanthae*	fruits / Germany	LZ 2010-502	HQ414587	–	–	Roensch et al. (2010)
*X.palmicola*	fruits / New Zealand	PDD 604	GU322436	GQ495929	GQ844822	Hsieh et al. (2010)
*X.phyllocharis*>	dead leaves / French West	HAST 528	GU322445	GQ495938	GQ844832	Hsieh et al. (2010)
*X.plebeja*	trunk / China Taiwan	HAST 91122401	GU324740	GQ502689	GQ848353	Hsieh et al. (2010)
*X.polymorpha*	wood / USA	JDR 1012	GU322460	GQ495954	GQ848343	Hsieh et al. (2010)
*X.polymorpha*	Stump / Germany	M:M-0125909	FM164944	–	–	Persoh et al. (2009)
***X.polysporicola***	**fallen leaves of *Polysporahainanensis* / China Haina**n	**FCATAS 848 (HT)**	MZ005592	**MZ031976**	**MZ031980**	**This study**
***X.polysporicola***	**fallen leaves of *Polysporahainanensis* / China Hainan**	**FCATAS 849**	**MZ005591**	**MZ031977**	**MZ031981**	**This study**
*X.regalis*	log of *Ficusracemose* / India	HAST 920	GU324745	GQ502694	GQ848358	Hsieh et al. (2010)
*X.schweinitzii*	bark / China Taiwan	HAST 92092023	GU322463	GQ495957	GQ848346	Hsieh et al. (2010)
X.siculaf.major	fallen leaves / China Taiwan	HAST 90071613	GU300081	GQ478216	GQ844794	Hsieh et al. (2010)
*Xylaria* sp. 6	fallen leaves of *Tibouchinasemidecandra* / USA	JDR 258	GU300082	GQ478217	GQ844795	Hsieh et al. (2010)
*X.striata*	branch / China	HAST 304	GU300089	GQ478224	GQ844803	Hsieh et al. (2010)
*X.tentaculata*	leaf litter or wood / Korea	KA12-0530	KM077162	–	–	Kim et al. (2016)
*X.tentaculata*	leaf litter or wood / Korea	KA13-1324	KM077163	–	–	Kim et al. (2016)
*X.tentaculata*.	leaf litter or wood / Korea	KA13-1325	KM077164	–	–	Kim et al. (2016)
*X.venosula*	twigs / USA	HAST 94080508	EF026149	EF025617	GQ844806	Hsieh et al. (2010)
*X.venustula*	bark / China Taiwan	HAST 88113002	GU300091	GQ487699	GQ844807	Hsieh et al. (2010)
*X.xylarioides*	wood / Iran	GUM 1151	KP218909	–	–	Hashemi et al. (2015)
*Hypoxylonfragiforme*	bark / France	HAST 383	JN979420	AY951720	–	Hsieh et al. (2005)
*Camilleaobularia*	– / Puerto Rico	ATCC 28093	KY610384	KX271243	–	Wendt et al. (2018)

### ﻿Phylogenetic analyses

The molecular phylogeny was inferred from a combined dataset of ITS, TUB and RPB2 sequences. The sequences retrieved from open databases originated from Hsieh et al. (2005), Persoh et al. (2009), Hsieh et al. (2010), Roensch et al. (2010), Hashemi et al. (2015), Kim et al. (2016), Ma and Li (2018) and Wendt et al. (2018) (Table 1). *Hypoxylonfragiforme* (Pers.) J. Kickx f. and *Camilleaobularia* (Fr.) Læssøe, J.D. Rogers & Lodge were selected as outgroup taxa. Sequences were aligned using the MAFFT online (http://mafft.cbrc.jp/alignment/server/). Alignments were optimised manually in BioEdit 7.0.5.3 (Hall 1999).

A combined matrix of ITS-RPB2-TUB and ITS-exons of TUB and RPB2 were used to construct phylogenetic analysis by two methods including maximum likelihood (ML) and Bayesian Inference (BI) analysis, respectively. ML tree generation and bootstrap analyses were performed via the programme RAxML7.2.6 (Stamatakis 2006) running 1000 replicates combined with a ML search. Bayesian analysis was performed with MrBayes 3.1 (Huelsenbeck and Ronquist 2005) implementing the Markov Chain Monte Carlo (MCMC) technique and parameters predetermined by MrModeltest 2.3 (Nylander 2004).

## ﻿Results

### ﻿Molecular phylogeny

This study used genetic sequences of 57 species, including 69 ITS sequences, 57 TUB sequences and 54 RPB2 sequences. We applied two tree construction methods to improve the reliability of the results.

After the alignment sequence was adjusted using MAFFT, the ITS alignment, shown in BioEdit 7.0.5, consisted of 778 character positions, 2219 in the TUB alignment and 1241 in the RPB2 alignment. After curing, the constructed multigene alignment (MGA) consisted of 3138 characters (523 of which were derived from the ITS alignment, 1550 from TUB alignment, 1065 from RPB2 alignment). Of the MGA, 1354 characters were considered parsimony-informative.

**Figure 1.**
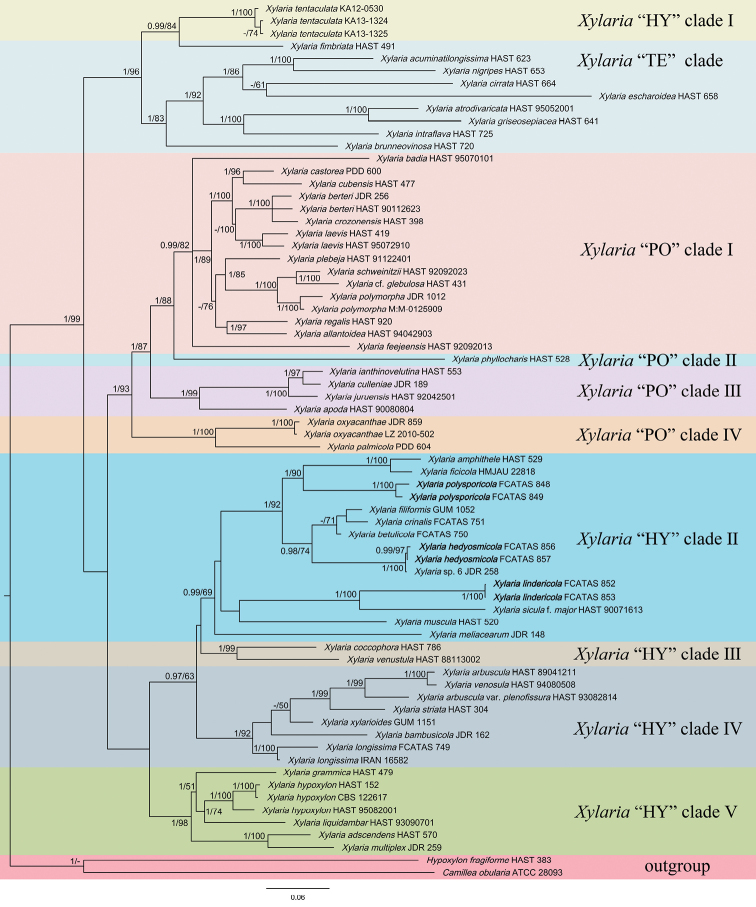
Phylogenetic tree of *Xylaria* based on multigene alignment of ITS-TUB-RPB2 in the Bayesian analysis. Bayesian posterior probabilities (≥ 0.95, before the slash markers) and RaxML bootstrap values (≥ 50, after the slash markers) are shown. Different clades are indicated as coloured blocks.

The analysis results show that the phylogenetic tree, generated by ML in RAxML7.2.6, is basically the same as that generated by BI in MrBayes 3.1. Topology of the phylogenetic analyses, based on ITS-RPB2-TUB and ITS-exons of TUB and RPB2, have no significant conflicts. Only the BI tree is shown in Figure 1 with Bayesian posterior probabilities ≥ 0.95 and ML bootstrap values ≥ 50% labelled along the branches. The phylogenetic tree showed that *X.hedyosmicola* is clustered with *Xylaria* sp. 6, *X.polysporicola* is clustered with *X.amphithele* F. San Martín & J.D. Rogers and *X.ficicola* Hai X. Ma, Lar.N. Vassiljeva & Yu Li, *X.lindericola* is clustered with X.siculaPass. & Beltr.f.major Ciccarone, but were separated from other species, as well as from each other.

## ﻿Taxonomy

### ﻿ Xylaria hedyosmicola

Taxon classificationFungiXylarialesXylariaceae

Hai X. Ma & X.Y. Pan sp. nov.

12087348-54F1-5006-A915-68982B5B3556

839780

Figure 2

#### Diagnosis.

Differs from *X.vagans* by its stromata without a black rhizomorphoid mycelium connecting dead leaves, larger ascospores and tubular to slightly urn-shaped apical apparatus. Differs from *X.betulicola* by its smaller stromta and larger ascospores.

#### Typification.

China. Hainan Province, Lingshui County, Diaoluoshan Natural Reserve, on fallen leaves of *Hedyosmumorientale* (Chloranthaceae), 31 December 2020, Haixia Ma (holotype, FCATAS 856).

#### Etymology.

“***hedyosmicola***” refers to the growth on leaves of *Hedyosmumorientale*.

#### Teleomorph.

*Stromata* upright, solitary to cespitose, thread-like, unbranched or occasionally branched once at top, 2–5.5 cm total length; with a long sterile filiform apex up to 0.5–3 cm long; fertile part 3–17 mm long × 0.5–1 mm diam., usually consisting of closely packed or scattered perithecia; stipe 8–18 mm long × 0.1–0.5 mm diam., glabrous, finely longitudinally striate, the base slightly swollen; surface roughened, with half-exposed to fully exposed perithecial contours and wrinkles. Externally black, interior white. Texture soft. *Perithecia* subglobose, 200–470 µm diam. *Ostioles* papillate, 11–22 µm diam. *Asci* with eight ascospores arranged in uniseriate manner, cylindrical, 105–160 µm total length, the spore-bearing parts 70–100 µm long × 8–12 µm broad, the stipes 25–70 µm long, with apical apparatus bluing in Melzer’s reagent, tubular to slightly urn-shaped, 2.5–4.8 µm high × 2.5–3.5 µm broad. *Ascospores* brown, unicellular, ellipsoid-inequilateral, with narrowly rounded ends, smooth, (12–)13–15(–16.7) × (6–) 6.5–7.5 (–8.5) µm (M = 14 × 7 µm, n = 60), straight to slightly sigmoid germ slit spore-length or almost spore-length, with a slimy sheath on ventral side swollen at both ends to form rounded non-cellular appendages visible in Indian ink.

**Figure 2.**
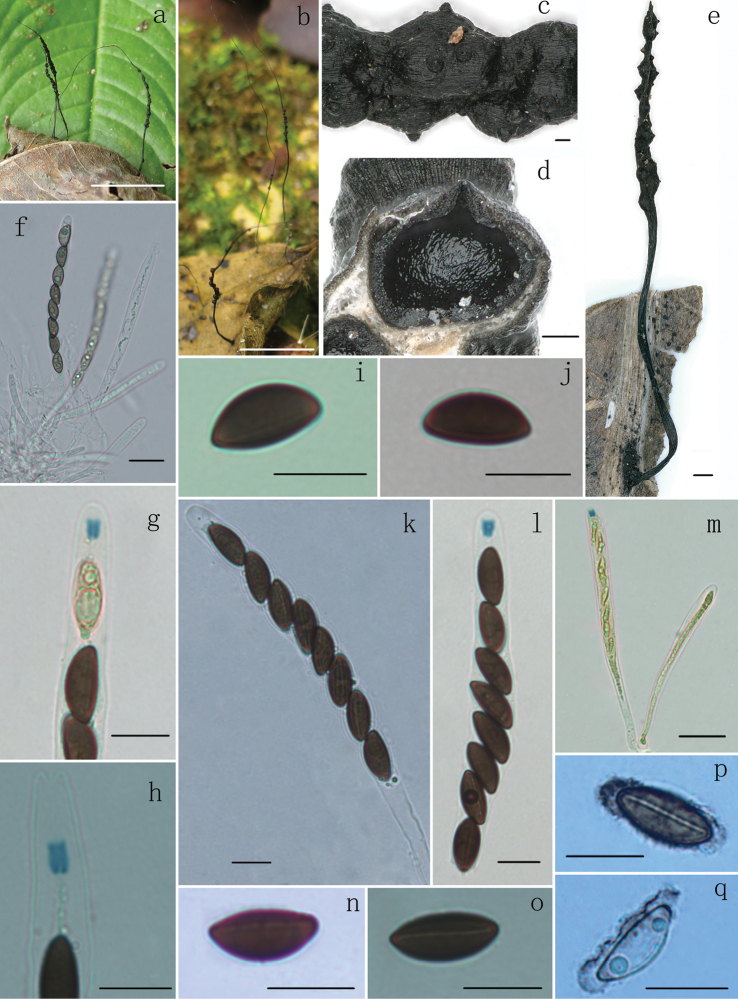
*Xylariahedyosmicola* (FCATAS 856, holotype) **a, b, e** stromata on leaves (b, FCATAS 857) **c** stromatal surface **d** section through stroma, showing a perithecium **f** immature asci in water **g, h** ascal apical ring in Melzer’s reagent **i, j** ascospores in Melzer’s reagent **k** ascus in 1% SDS **l, m** asci and ascal apical ring in Melzer’s reagent **n** ascospore in Melzer’s reagent showing straight germ slit **o** ascospore in Melzer’s reagent showing slightly sigmoid germ slit **p, q** ascospore showing a slimy sheath and non-cellular appendages in India ink. Scale bars: 1 cm (**a, b**); 0.1 mm (**c, d**); 0.5 mm (**e**); 20 µm (**f, m**); 10 µm (**g–l, n–q**).

#### Additional specimen examined.

China. Hainan Province, Lingshui County, Diaoluoshan Natural Reserve, on fallen leaves of *Hedyosmumorientale*, 31 December 2020, Haixia Ma (FCATAS 857).

#### Remarks.

*Xylariahedyosmicola* closely resembles *X.vagans* Petch by sharing thread-like or long hair-like stromata bearing closely packed or scattered perithecia with a long sterile filiform apex. *Xylariavagans* was originally described and illustrated by Petch (1915) from Sri Lanka. However, based on comparisons of the descriptions and illustrations, there were some differences between the two species. *Xylariahedyosmicola* has larger sporiferous part of asci (70–100 µm × 8–12 µm) with tubular to slightly urn-shaped apical apparatus bluing in Melzer’s reagent, brown and larger ascospores with straight (Fig. 2n and p) to slightly sigmoid germ slit (Fig. 2o), with narrowly rounded ends and a slimy sheath on ventral side swollen at both ends to form rounded non-cellular appendages, while *X.vagans* has a black rhizomorphoid mycelium connecting dead leaves, smaller sporiferous part 68–72 µm × 6 µm and black-brown, cymbiform, smaller ascospores 9–12 × 5–6 µm, with broadly rounded ends and is without apical apparatus, germ slit and sheath or appendages (Petch 1915). Unfortunately, the molecular sequences of *X.vagans* from Sri Lanka were not available.

*Xylariabetulicola* Hai X. Ma, Lar.N. Vassiljeva & Yu Li is similar to *X.hedyosmicola* in stromatal morphology, but differs in having larger stromata 3–7 cm, slightly smaller ascospores (11.5)12–14(15) × 5–6 µm, without sheath or appendages (Ma and Li 2018). In the phylogenetic tree, *X.hedyosmicola* formed a fully supported clade with *Xylaria* sp. 6 from Hawaiian Islands, USA (Hsieh et al. 2010). Although there are no descriptions on *Xylaria* sp. 6 in the study of Hsieh et al. (2010), we suspected that it is conspecific with *X.hedyosmicola*. The sequences comparison showed that there are 98.7%, 99% and 99.9% maximal percentage identities, respectively in ITS, TUB and RPB2 between *X.hedyosmicola* (FCATAS 856) and *Xylaria* sp. 6 from USA (JDR 258).

### ﻿ Xylaria lindericola

Taxon classificationFungiXylarialesXylariaceae

Hai X. Ma & X.Y. Pan sp. nov.

8273E502-A2D0-5CBF-BBDA-8B1CA1211B59

839554

Figure 3

#### Diagnosis.

Differs from X.siculaf.major by its subglobose stromata without a long sterile apex, larger ascospores and host plant. Differs from *X.hypsipoda* by its black stromata, glabrous stipes and smaller apical apparatus.

#### Typification.

China. Hainan Province, Lingshui County, Diaoluoshan Natural Reserve, on fallen leaves of *Linderarobusta* (Lauraceae), 31 December 2020, Haixia Ma (holotype, FCATAS 852).

#### Etymology.

“***lindericola***” refers to the growth on leaves of *Linderarobusta*.

#### Teleomorph.

*Stromata* upright or prostrate, solitary to cespitose, unbranched or branched once or more at stipe, 3–26 cm total length; fertile part subglobose on long filiform stipes, 0.1–0.4 cm diam., the stipe 3–25 cm long × 0.1–1 mm diam., glabrous, finely longitudinally striate, the base slightly swollen; surface roughened by wrinkles and barely exposes perithecial contours. External black, interior white. Texture soft. *Perithecia* subglobose, 300–550 µm diam. *Ostioles* black, papillate. *Asci* with eight ascospores in uniseriate manner, cylindrical, 105–165 µm total length, the spore-bearing parts 65–115 µm long × 7.5–10.5 µm broad, the stipes 25–65 µm long, with apical apparatus bluing in Melzer’s reagent, tubular to urn-shaped, 3.9–5.5 µm high × 3–5 µm broad. *Ascospores* brown, unicellular, ellipsoid-inequilateral, with slightly narrowly rounded ends, aberrant ascospores with strongly pinched or beaked ends, smooth, (12.5–)13.5–15.5(–18) × (7–) 7.5–8.5 (–9.5) µm (M = 14.8 × 8 µm, n=60), with straight germ slit spore-length, without sheath or appendages visible in India ink.

#### Additional specimen examined.

China. Hainan Province, Lingshui County, Diaoluoshan Natural Reserve, on fallen leaves of *Linderarobusta*, 31 December 2020, Haixia Ma (FCATAS 853).

#### Remarks.

*Xylarialindericola* is distinguished by its subglobose fertile part of stroma on a long filiform stipe and growing on fallen leaves of *Linderarobusta*. The species is somewhat similar to X.siculaf.major in morphology of stromatal fertile part. However, X.siculaf.major has stromata with long sterile apex, slightly smaller ascospores 9–13(–15) × (3–) 4.5–6 (–7) µm and grows on dead *Olea* leaves (Ciccarone 1947; Graniti 1959; Fournier 2014). In the phylogenetic tree, *X.lindericola* formed a fully supported clade with X.siculaf.major (Figure 1).

*Xylariahypsipoda* Massee is similar to *X.lindericola* by sharing globose stromata and ascospores dimensions, but differs in having stromata with whitish scales, hairy stipes and urn-shaped, slightly larger apical apparatus 5–8 µm high × 2.9–5 µm broad (Rogers et al. 1987).

*Xylariaficicola* resembles *X.lindericola* in stromatal morphology, but differs in having strongly exposed perithecial mounds of stromatal surface, larger ascospores (16–) 17.5–21(–22.7) × 6.5–8.5 µm with conspicuous hyaline noncellular appendage and grows on fallen leaves and petioles of *Ficusauriculata* (Ma et al. 2011). *Xylariaheloidea* Penz. & Sacc. from Indonesia is somewhat similar to *X.lindericola* in stromatal morphology, but the former has obconical, convex stromatal top, larger ascospores (14.5–) 15.5–18(–19) × (5–)5.5–6.5(–7) µm (16.7 × 6.1 µm), with a hyaline sheath swelling at both ends to form non-cellular appendages and grows on fallen fruits, twigs, petioles, and leaves of various plants (Ju et al. 2018).

*Xylariacomosa* (Mont.) Fr. and *X.clusiae* K.F. Rodrigues, J.D. Rogers & Samuels are also somewhat similar to *X.lindericola* in stromatal morphology. However, *X.comosa* has larger ascospores (21)–26–40 × 7–11 µm and larger apical ring 10.5 µm high × 7.5 µm broad (Dennis 1956) and *X.clusiae* has smaller stromata 1–3.5 cm, ascospores broadly ovoida1 to nearly globose (11.6–)12.8–16.7(–18) × 8–15 µm, with colorless appendage at one end (Samuels and Rogerson 1990).

**Figure 3.**
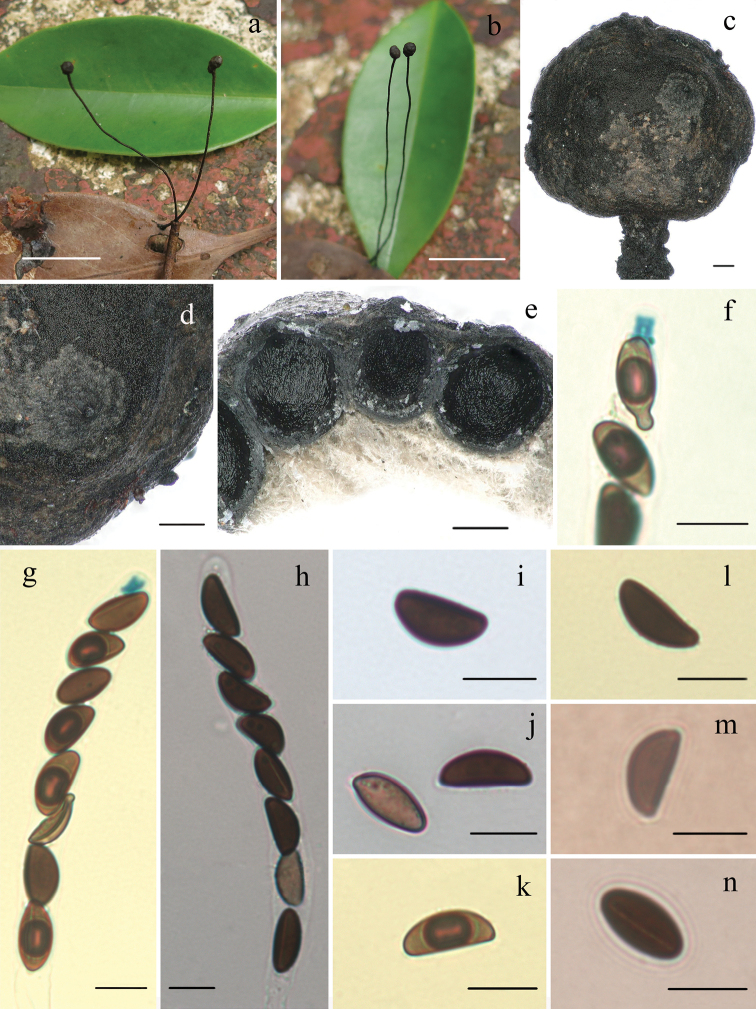
*Xylarialindericola* (FCATAS 852, holotype) **a, b** stromata on leaves **c** fertile part of stroma **d** stromatal surface **e** section through stroma, showing perithecia **f** ascal apical ring and ascospores with beaked ends in Melzer’s reagent **g** ascus and ascal apical ring in Melzer’s reagent **h** ascus in water **i, j** ascospores in water **k, l** ascospore in Melzer’s reagent **m** ascospore in India ink **n** ascospore in 1% SDS showing germ slit. Scale bars: 1.5 cm (**a, b**); 0.2 mm (**c–e**); 10 µm (**f–n**).

### ﻿ Xylaria polysporicola

Taxon classificationFungiXylarialesXylariaceae

Hai X. Ma & X.Y. Pan sp. nov.

697BC6F2-DC69-5FDC-BDE5-B67B0EAB0FAC

839552

Figure 4

#### Diagnosis.

Differs from *X.phyllocharis* by its half-exposed to fully exposed perithecial contours, the fertile part cylindrical and larger perithecia. Differs from *X.phyllophila* by its smaller ascospores. Differs from *X.amphithele* by its cylindrical stromata.

#### Typification.

China. Hainan Province, Lingshui County, Diaoluoshan Natural Reserve, on fallen leaves of *Polysporahainanensis* (Theaceae), 31 December 2020, Haixia Ma (holotype, FCATAS 848).

#### Etymology.

“***polysporicola***” refers to the growth on leaves of *Polysporahainanensis*.

#### Teleomorph.

*Stromata* solitary, upright or prostrate, cylindrical, unbranched or occasionally branched, 1–4 cm total length, with acute sterile apex up to 2 mm long; fertile part 2–15 mm long × 0.5–1.6 mm diam., usually consists of closely packed perithecia and occasionally with scattered perithecia; the stipe 5–30 mm long × 0.3–1 mm diam., glabrous, finely longitudinally striate, the base slightly swollen; surface roughened, with half-exposed to fully exposed perithecial contours and wrinkles. Externally black, interior white. Texture soft. *Perithecia* subglobose, 0.4–0.6 mm diam. *Ostioles* papillate. *Asci* with eight ascospores arranged in uniseriate manner, cylindrical, 115–185 µm total length, the spore-bearing parts 75–100 µm long × 6.5–9 µm broad, the stipes 30–90 µm long, with apical apparatus bluing in Melzer’s reagent, inverted hat-shaped or urn-shaped, 2.5–4.5 µm high × 2–3.2 µm broad. *Ascospores* brown to dark-brown, unicellular, ellipsoidal-inequilateral, with broadly rounded ends, one end slightly pinched sometimes, smooth, (11.5–)12.5–14.5(–15) × 5.5–8 µm (M = 13.2 × 6.4 µm, n=60), with straight germ slit slightly less than spore-length, a slimy sheath or non-cellular appendages visible occasionally in Indian ink.

#### Additional specimens examined.

China. Hainan Province, Lingshui County, Diaoluoshan Natural Reserve, on fallen leaves of *Polysporahainanensis*, 31 December 2020, Haixia Ma (FCATAS 849); 5 July 2019, Haixia Ma (FCATAS 850 & 851).

#### Remarks.

*Xylariapolysporicola* is morphologically similar to *X.phyllocharis* Mont. However, *X.phyllocharis* has fully immersed perithecia, the fertile part with peg-like structures and smaller perithecia 0.2–0.3 mm diam (San Martín and Rogers 1989; Fournier et al. 2020). *Xylariapolysporicola* is similar to *Xylaria* sp. (80082005) from Taiwan in stromatal morphology, but the latter has slightly smaller stroma (11–14 mm total length × 1 mm diam. vs. 10–40 mm total length × 0.5–1.6 mm diam.), hard texture, slightly larger ascospores 13.5–16.5 × 5–6 µm, with narrowly rounded ends (Ju and Rogers 1999). *Xylariaphyllophila* Ces. somewhat resembles *X.polysporicola* in stromatal morphology, but the former has larger ascospores 20 × 10 µm (Cooke 1883).

**Figure 4.**
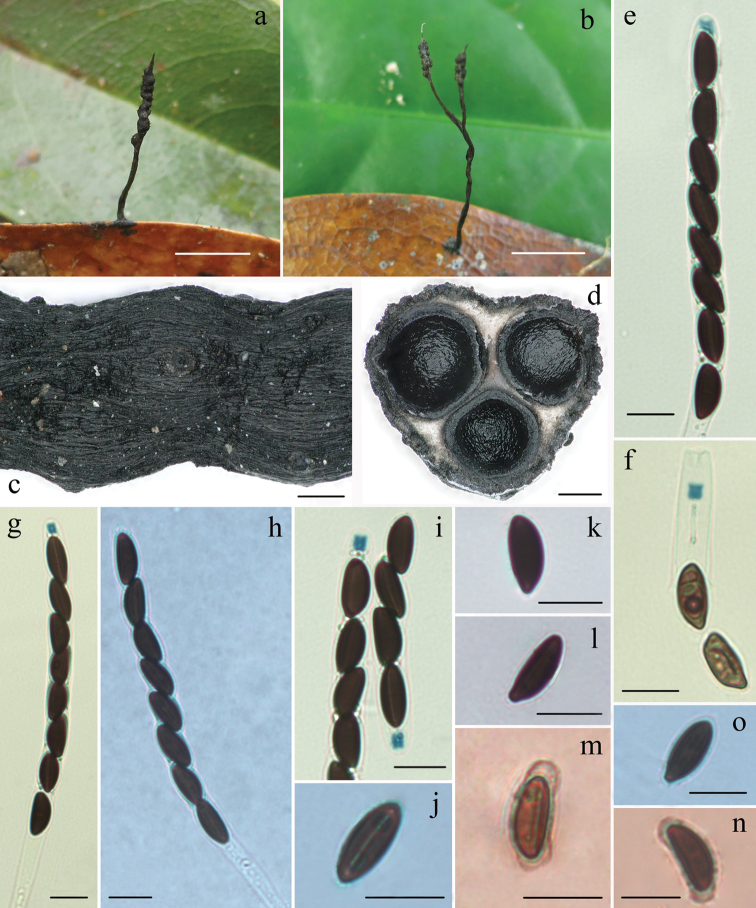
*Xylariapolysporicola* (FCATAS 848, holotype) **a, b** stromata on leaves (b, FCATAS 851) **c** stromatal surface **d** section through stroma, showing perithecia **e, g** asci and ascal apical ring in Melzer’s reagent **f, i** ascal apical ring in Melzer’s reagent **h** asci in black India ink **j** ascospore with germ slit in 1% SDS **k, l** ascospore in water **m, n** ascospore showing a slimy sheath and non-cellular appendages in India ink (FCATAS 850) **o** Ascospore in 1% SDS. Scale bars: 1 cm (**a, b**); 0.2 mm (**c, d**); 10 µm (**e–o**).

*Xylariapolysporicola* is somewhat similar to *X.amphithele* F. San Martín & J.D. Rogers in shape and size of apical apparatus and ascospores. However, *X.amphithele* has globose to conical stromata with 3–4 to 20 naked perithecia (San Martín and Rogers 1989). In the phylogenetic tree, *X.polysporicola* formed a lineage close to *X.amphithele* and *X.ficicola*, but is distant from *X.phyllocharis*.

## ﻿Discussion

We included ten *Xylaria* species on fallen leaves in the phylogenetic analyses of the present study. Except for *X.phyllocharis*, the other nine studied species formed a monophyletic clade with two wood-inhabiting species, *X.muscula* Lloyd and *X.crinalis* Hai X. Ma, Lar. N. Vassiljeva & Yu Li, in our phylogenetic tree (Figure 1). In China, only three species have been previously reported with molecular evidence: *X.ficicola* from tropical Yunnan, X.siculaf.major from tropical Taiwan and *X.betulicola* from temperate Jilin (Ma and Li 2018). Within the clade, *X.meliacearum*, associated with petioles and infructescence of *Guareaguidonia*, formed a separate branch from other *Xylaria* species on other leaves. In Hsieh et al. (2010), *X.phyllocharis* grouped with the wood-inhabiting *Xylaria* species, which did not reveal any contradictions in our tree. Three species, *X.polysporicola*, *X.amphithele* and *X.ficicola* formed a highly supported clade. Morphologically, these species have some similar features, such as ascospores with slimy sheath or non-cellular appendages, inverted hat-shaped or urn-shaped apical apparatus (San Martín and Rogers 1989; Ma et al. 2020). As *Xylariahedyosmicola* formed a fully supported clade with *Xylaria* sp. 6, the two species should be the same, based on the ITS-TUB-RPB2 (Hsieh et al. 2010). *Xylarialindericola*, on leaves of *Linderarobusta* formed a sister lineage to X.siculaf.major on unknown fallen leaves with high bootstrap value 100%. *Xylariamuscula*, growing on dead branches, formed a weakly supported branch with *X.lindericola* and X.siculaf.major associated with fallen leaves in our tree. This may be because our phylogenetic analysis did not include more taxa related to *X.muscula*.

Until now, ten taxa, *X.betulicola*, *X.diminuta* F. San Martín & J.D. Rogers, *X.ficicola*, *X.foliicola* G. Huang & L. Guo, *X.hainanensis* Y.F. Zhu & L. Guo, *X.hedyosmicola*, *X.jiangsuensis* G. Huang & L. Guo, *X.lindericola*, *X.polysporicola* and X.siculaf.major have been found on fallen leaves in China (Hsieh et al. 2010; Ma et al. 2011; Zhu and Guo 2011; Huang et al. 2014, 2015; Ma and Li 2018). Amongst these species, *X.diminuta*, originally reported from Mexico, was found in Yunnan province of China in 2013 (Huang et al. 2014). Xylariasiculaf.major was first described from Sicily in 1878 and then found in Spain, Kenya, Sardinia, and Taiwan province of China (Hsieh et al. 2010; Fournier 2014). Unfortunately, except for the three species in this study, the molecular data of the other *Xylaria* species from China were not available. We anticipate that additional species of *Xylaria* on fallen leaves will be discovered as more studies are conducted.

### ﻿Key to species of *Xylaria* on fallen leaves in China

**Table:** 

1	Stromata with rounded fertile apices	**2**
–	Stromata with acute sterile apices	**3**
2	Stromata associated with leaves and petioles of *Ficusauriculata* (Moraceae), ascospores (16–)17.5–21(–22.7) × 6.5–8.5 µm	***X.ficicola***
–	Stromata associated with leaves of *Linderarobusta* (Lauraceae), ascospores (12.5–)13.5–15.5(–18) × (7–)7.5–8.5(–9.5) µm	***X.lindericola***
3	Stipes tomentose	***X.hainanensis***
–	Stipes glabrous	**4**
4	Fertile part subglobose	**X.siculaf.major**
–	Fertile part not subglobose	**5**
5	Stromata cylindrical	**6**
–	Stromata filiform	**8**
6	Ascospores (5.5–)6–8 × 3–3.5(–4) µm	***X.diminuta***
–	Ascospores length > 8.5 µm	**7**
7	Stromata with conspicuous perithecial contours, ascospores (11.5–)12.5–14.5(–15) × 5.5–8 µm	***X.polysporicola***
–	Stromata with inconspicuous perithecial contours, ascospores (8.5–)9–11 × 4–6 µm	***X.foliicola***
8	Ascospores 16.5–20(–21.5) × 4–5(–6) µm	***X.jiangsuensis***
–	Ascospores length < 16.5 µm	**9**
9	Stromata associated with leaves of *Betula* (Betulaceae), ascospores (11.5)12–14(15) × 5–6 µm, with a straight germ slit, without appendages visible in India ink	***X.betulicola***
–	Stromata associated with leaves of *Hedyosmumorientale* (Chloranthaceae), ascospores (12–)13–15(–16.7) × (6–)6.5–7.5(–8.5) µm, with straight to slightly sigmoid germ slit, with appendages visible in Indian ink	***X.hedyosmicola***

## Supplementary Material

XML Treatment for Xylaria hedyosmicola

XML Treatment for Xylaria lindericola

XML Treatment for Xylaria polysporicola
